# Co-Cultivation with *Eichhornia crassipes* Enhances Growth and Ovarian Development of *Micropterus salmoides*

**DOI:** 10.3390/ijms27010398

**Published:** 2025-12-30

**Authors:** Lin Zhang, Jiahao Liu, Jiawen Hu, Nailin Shao, Yi Sun, Jiahui Xiao, Zhijuan Nie, Pao Xu

**Affiliations:** 1 Freshwater Fisheries Research Center (FFRC), Chinese Academy of Fishery Sciences (CAFS), Wuxi 214081, China; zhanglin@ffrc.cn (L.Z.);; 2 Wuxi Fisheries College, Nanjing Agricultural University, Wuxi 214081, China; liujh@stu.njau.edu.cn (J.L.);

**Keywords:** largemouth bass, watery hyacinth, transparency, ovarian development

## Abstract

The growth and development of aquaculture organisms are significantly influenced by environmental variations shaped by different aquaculture systems. In this study, a 90-day controlled experiment was conducted to compare two pond culture setups for largemouth bass: with water hyacinth co-planted (FM group) and without (M group). As this experiment progressed, the FM group exhibited significantly superior water quality (*p* < 0.05) compared to the M group across multiple parameters, including total nitrogen (TN), total phosphorus (TP), ammonia nitrogen (NH_3_-N), dissolved oxygen (DO) and transparency, among which, the difference in transparency was especially evident (*p* < 0.001). Subsequently, by 90 days, the fish body weight, condition factor, and gonadosomatic index (GSI) were significantly higher in the FM group than in the M group, with the GSI difference being particularly pronounced (*p* < 0.001). While the GSI of M group fishes ranged exclusively from 0.01 to 0.02 (M1), the FM group displayed a much-expanded GSI range of 0.01–0.06, with 21.4% at 0.01–0.02 (FM1), 48.1% at 0.02–0.03 (FM2), and 30.5% at 0.03–0.06 (FM3). Accordingly, omics analyses of ovarian tissues were conducted between the control (M1) and the high-performing groups (FM2 and FM3). The analyses identified significant enrichment of the glycerophospholipid metabolic pathway and a marked upregulation of the *Mettl3* gene (log2FC = 12.59) in the FM2 and FM3 than the M1 group, and both the pathway and the *Mettl3* gene were actively involved in growth, reproductive processes, and oocyte maturation. Given that water transparency was the most markedly improved parameter, our results indicate that it may be a key driver in upregulating ovarian glycerophospholipid metabolism and *Mettl3* expression in largemouth bass, thereby promoting better growth and ovarian development.

## 1. Introduction

Native to the southeastern United States [1], *Micropterus salmoides* (largemouth bass, hereafter LMB) was first introduced to mainland China in 1983, mainly for aquaculture [2]. It has since become a critically important high-value species in Chinese aquaculture due to its advantageous traits in growth rate, taste, nutritional value, intermuscular spines, feed utilization, and environmental adaptability [3,4,5]. This expansion was dramatically accelerated by the breakthrough in specialized formulated feed around 2017, resulting in a 2024 yield of 938,000 tons [6], more than double the 347,300 tons recorded in 2016.

While new farming models are being explored [7], intensive pond monoculture continues to dominate LMB production in China. This reliance on high-density cultivation, however, exacerbates challenges in preserving pond water quality and ecological stability. A major issue is nutrient overloading, driven by elevated inputs of exogenous feed and the accumulation of nutrient-rich residues on the pond bottom. This eutrophication often triggers harmful cyanobacterial blooms and subsequent hypoxic events, severely compromising fish health, growth performance, and ultimately, food safety and quality [8,9]. In addition, the reproductive system is particularly vulnerable to such degraded water conditions. Evidence from wild fish populations shows that water contamination can lead to the prevalence of gonadal diseases, as observed in mullets [10]. Controlled laboratory studies further demonstrate that exposure to harmful cyanobacteria (e.g., *Oscillatoriaceae*) can disrupt sex hormone levels and delay gametogenesis in zebrafish [11]. More far-reaching, research indicates that hypoxia can induce transgenerational harm: Epigenetic changes in the sperm of exposed males (F0) led to reduced sperm motility and quantity, persisting into two subsequent unexposed generations [12]. These findings collectively underscore that ensuring a safe water environment to prevent these cascading risks is fundamental to the success of aquaculture, especially in intensive systems.

Against this background, ecological aquaculture, which simulates natural habitats and prioritizes ecosystem sustainability, has become a strategic focus [13]. A common practice is the co-cultivation of large aquatic plants with fish. *Eichhornia crassipes* (water hyacinth) is one such macrophyte widely used for water quality enhancement. Water hyacinth has both rapid growth and fast biomass accumulation. Its well-proved phytoremediation capacity effectively removes excessive nutrients and contaminants, such as organics, heavy metals, and suspended solids, thereby mitigating water eutrophication and enhancing water transparency, among others [14,15,16,17,18]. Additionally, its physical presence mitigates algal blooms by competing for light and nutrients while contributing to dissolved oxygen (DO) levels in the water [19,20,21]. Notably, water quality indicators such as transparency, DO, and nutrient load (e.g., total nitrogen and total phosphorous, TN/TP) are established environmental factors influencing fish growth and development [22,23]. Among these, light conditions—largely determined by water transparency—have been validated as a key factor regulating the reproductive cycle in farmed fish, governing the initiation and inhibition of gonadal development through neuroendocrine pathways [24]. In short, when properly managed, water hyacinth can help to maintain or even enhance water quality, thereby exerting a positive influence on fish growth and development. It is therefore reasonable to hypothesize that the controlled introduction of water hyacinth into LMB ponds could modulate fish growth and gonadal development by mediating water quality. To date, however, no studies have systematically investigated these potential effects or their underlying mechanisms in modulating LMB.

To address this gap, in this study, we established an ecological aquaculture model by introducing water hyacinth into largemouth bass ponds, contrasting it with a conventional model without water hyacinth. By monitoring and comparatively analyzing key water quality parameters, fish growth performance, and gonadal development traits across the two models—with a specific focus on molecular variations identified in ovarian tissue—we aimed to evaluate the efficacy of the water hyacinth-integrated model and explore the potential molecular mechanisms underlying the observed biological effects.

## 2. Results

### 2.1. Water Quality Parameters

**Figure 1:**
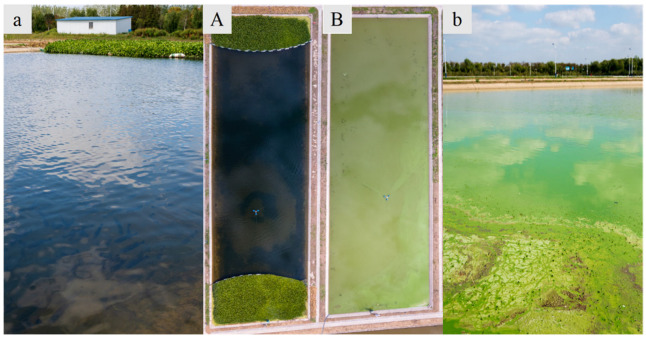
Water appearance of the experimental (FM) and control (M) ponds. (Note: Panels (**A**,**B**) show the panoramic views of FM and M ponds, respectively; panels (**a**,**b**) provide corresponding unilateral close-up views, respectively). The images were taken on Day 60 of this experiment.

The appearance of water in the experimental and control ponds is compared visually in Figure 1, with corresponding water quality parameters presented in Table 1. The control ponds exhibited a distinct cyanobacterial bloom, characterized by a blue-green color and extensive surface coverage (Figure 1). In contrast, the experimental ponds with water hyacinth maintained clearer, more translucent water, allowing for better visibility of fish and showing no signs of cyanobacterial proliferation. After 90 days of farming, water quality parameters differed significantly between groups (Table 1). The total nitrogen, nitrate nitrogen, ammonia nitrogen, and total phosphorus levels in the control groups were significantly higher than those in the experimental groups (*p* < 0.05). On the other hand, the water transparency value of the FM group ponds was recorded at 165.24 cm, dramatically greater than the 30.15 cm of the control group (*p* < 0.001) (Table 1). Given that water transparency was the most distinct parameter between the FM and M groups from the mid till the end of this experiment, it is likely a definitive factor underlying the subsequent disparities manifested in the fish.

### 2.2. Growth Index of Largemouth Basses

**Figure 2:**
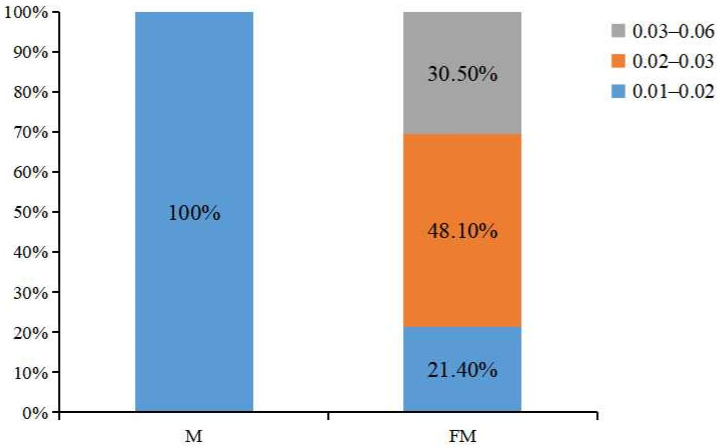
Proportion of largemouth basses with different gonadosomatic indices.

Throughout this experiment, no significant differences in body length or visceral body ratio were detected between the two groups (*p* > 0.05). However, by Day 90, the body weight, condition factor, and the gonadal indices of largemouth bass in the FM group were significantly higher than those of the M group (*p* < 0.05), among which the difference in gonad development (GSI) was particularly significant (*p* < 0.001) (Table 2). While all GSI values of the M group fell entirely within a narrow range of 0.01–0.02, the FM group exhibited a much wider GSI range of 0.01–0.06, with 21.4% of individuals in 0.01–0.02, 48.1% in 0.02–0.03, and 30.5% in 0.03–0.06 (Figure 2).

### 2.3. Morphological and Histological Observation

**Figure 3:**
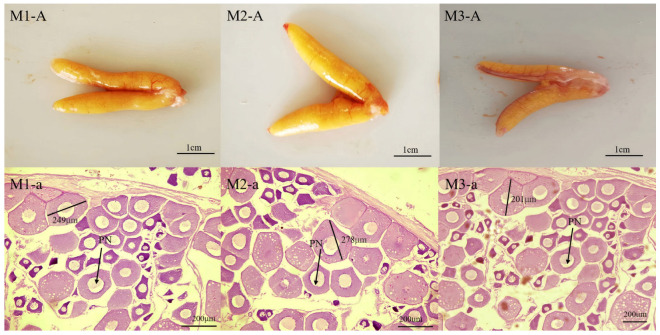
Appearance and histological observation of gonads of largemouth bass in M group (M1-A, M2-A, M3-A, ovaries of group M samples; M1-a, M2-a, M3-a, histological sections of the ovaries of group M samples; PN = perinucleolar oocytes).

**Figure 4:**
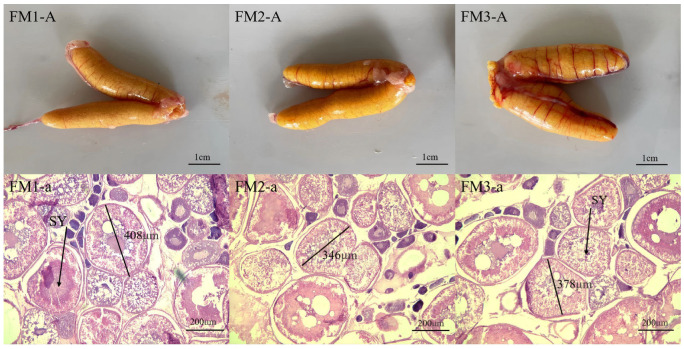
Appearance and histological observation of gonads of largemouth bass in FM group (FM1-A, FM2-A, FM3-A, ovaries of group FM samples; FM1-a, FM2-a, FM3-a, histological sections of the ovaries of group FM samples; SY = secondary yolk).

Figure 3 and Figure 4 show the gross morphology and histological sections of the gonads from group FM and group M, respectively, illustrating their appearance and development status. As is evident, the gonads of group M largemouth basses were thinner than those of group FM, and their germ cells were polygonal in shape, with a diameter of about 201–278 μm; most of the oocytes were perinucleolar oocytes. On the other hand, the gonads of FM largemouth basses were wider in shape and golden in color, with clear oocyte grains and a distinct size separation between developing and resting oocytes. Meanwhile, the oocytes developed synchronously, measuring between 346 and 408 μm in diameter. Most of the oocytes were secondary yolk.

### 2.4. Metabolomics Analysis

**Figure 5:**
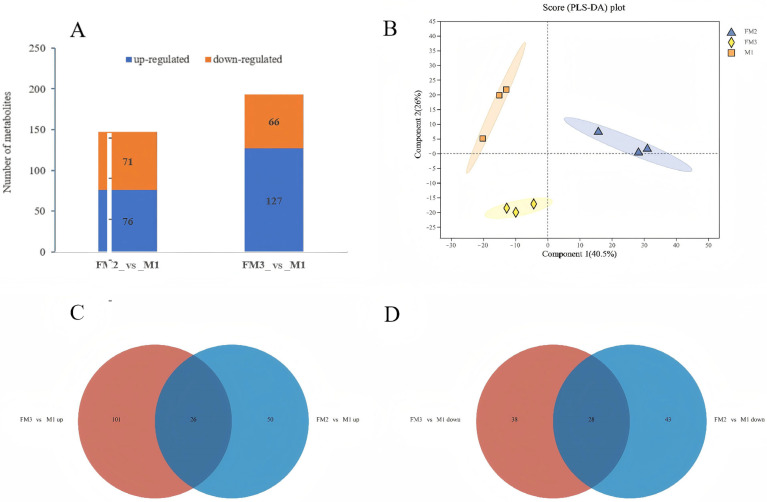
Comparative analysis of metabolites in FM3_vsVS_M1 and FM2_vs_M1 comparisons. (**A**) Differential metabolism histogram; (**B**) PLS-DA analysis chart; (**C**) Venn diagram of upregulated metabolites; and (**D**) Venn diagram of downregulated metabolites.

**Figure 6:**
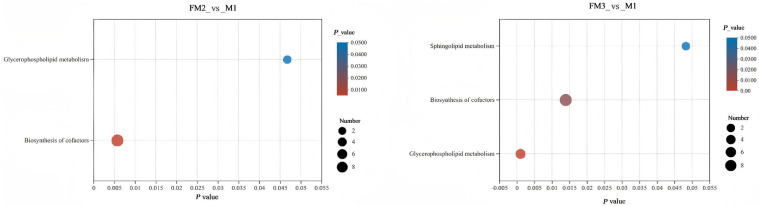
Pathway enrichment analysis of differential metabolites.

Using LC-MS to analyze the metabolite composition of gonad samples of M1, FM2, and FM3, a total of 6272 ion peaks were obtained, including 836 identifiable metabolites. Based on VIP > 1, *p* < 0.05, and |log2FC| ≥ 1, the comparison between samples FM3 and M1 revealed the highest number of differentially accumulated metabolites (DAMs), totaling 193 (127 DAMs upregulated and 66 DAMs downregulated). The comparison between FM2 and M1 resulted in 147 DAMs (76 DAMs upregulated and 71 DAMs downregulated) (Figure 5A). The Venn diagram reveals that FM3_vs_M1 and FM2_vs_M1 share 26 up-regulated differential metabolites and 28 downregulated ones (Figure 5C,D). The PLS-DA results indicate good reproducibility within the three gonad sample groups, with significant differences observed between the groups, reflecting notable metabolic variations among the different groups (Figure 5B). Pathway enrichment analysis shows that in FM3_vs_M1 comparison, such pathways were significantly enriched as glycerophospholipid metabolism, cofactor biosynthesis, and sphingolipid metabolism, while in FM2_vs_M1, enriched were glycerophospholipid metabolism and cofactor biosynthesis, with glycerophospholipid metabolism being the most enriched pathway in both cases (Figure 6).

### 2.5. Transcriptome Analysis and Metabolite-Transcriptome Co-Analysis

**Figure 7:**
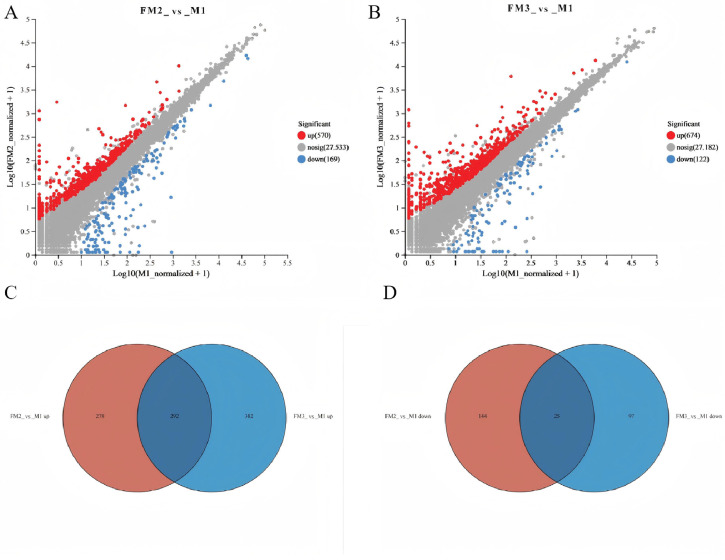
Comparative analysis of transcriptomes. (**A**,**B**) Volcano map; (**C**) Venn diagram of up-regulated differential genes; and (**D**) Venn diagram of downregulated differential genes among the three groups.

**Figure 8:**
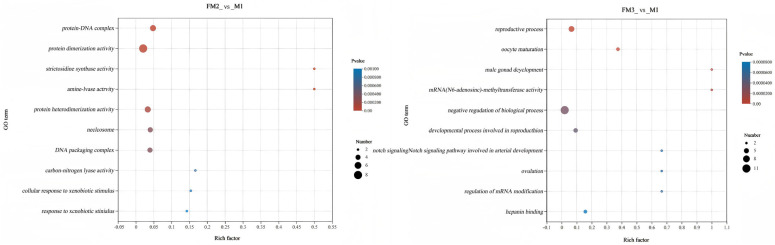
Pathway enrichment analysis of differential genes.

**Figure 9:**
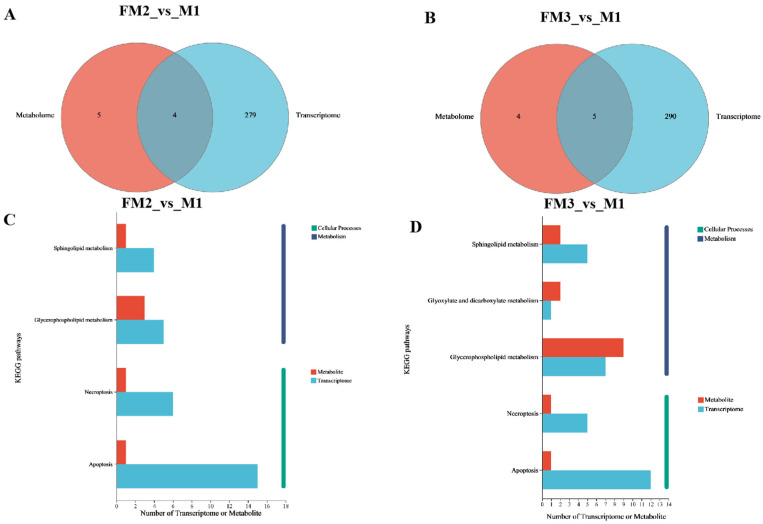
Pathway annotation analysis for integrated metabolomics and transcriptomic. (**A**,**C**) Venn diagram and (**B**,**D**) histogram.

Based on the criteria of |log2 fold change| ≥ 1 and *p* ≤ 0.05, a comparison between the gonadal tissue samples of the FM group and the M group identified 739 differentially expressed genes (DEGs) in FM2_vs_M1 (570 upregulated and 169 downregulated), while FM3_vs_M1 had 739 DEGs (674 upregulated and 122 downregulated) (Figure 7A,B). The Venn diagram revealed that the highest number of commonly upregulated DEGs (292) were shared between FM2_vs_M1 and FM3_vs_M1, accounting for 51% and 43% of the total upregulated DEGs in each comparison, respectively, whereas there were 25 commonly downregulated DEGs, representing 15% and 20% of the total downregulated DEGs in each comparison (Figure 7C,D). The top 10 enriched results of pathway enrichment analysis for differentially expressed genes are displayed in Figure 8. The differentially expressed genes in the FM3_vs_M1 group are also predominantly enriched in reproductive process, oocyte maturation, male gonad development, and mRNA (N6-adenosine)-methyltransferase activity (*p* ≤ 0.05). To further investigate the association between metabolites and genes in ovarian samples, a comprehensive analysis of metabolomics and transcriptomics was conducted. FM2_vs_M1 and FM3_vs_M1 identified four and five commonly annotated metabolic pathways, respectively, among which glycerophospholipid metabolism and sphingolipid metabolism play significant roles in ovarian development (Figure 9).

**Figure 10:**
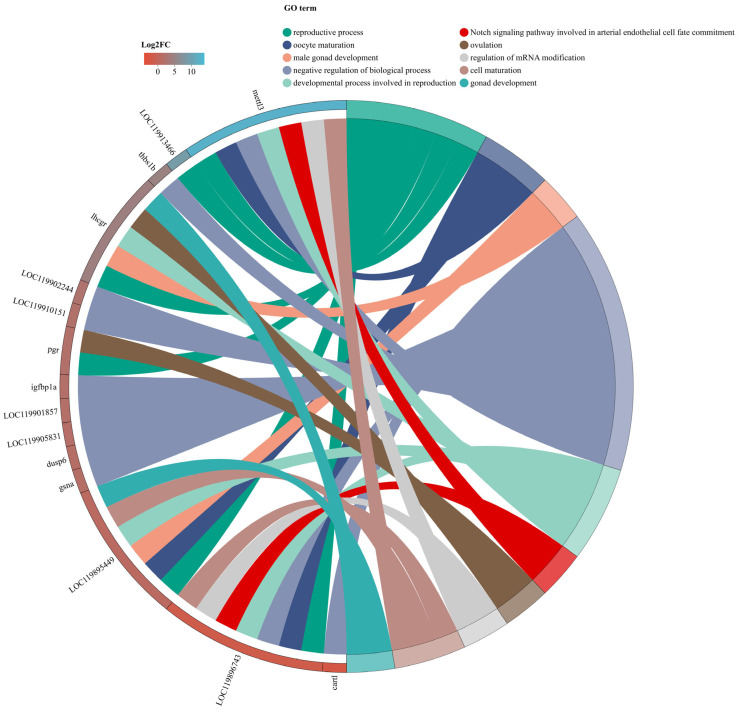
GO-enriched chordal plots of top 10 ranked overrepresented GO terms. The genes are linked to their assigned terms via colored ribbons. Genes are ordered according to the observed log-fold change (log2FC), which is displayed in decreasing intensity of red squares displayed next to the selected genes.

To enhance our understanding of the mechanisms underlying LMB gonadal development, we generated GO-enriched chordal plots (Figure 10) for the top ten most significantly enriched GO terms associated with the differentially expressed genes (DEGs) from the two most divergent counter groups, specifically FM3_vs_M1. The figure illustrates that the mettl3 gene, identified as an upregulated gene, exhibited the largest fold change in expression (log2FC = 12.59). This gene is associated with several biological processes, including reproductive processes, oocyte maturation, negative regulation of biological processes, developmental processes involved in reproduction, the Notch signaling pathway related to arterial endothelial cell fate commitment, regulation of mRNA modification, and cell maturation.

### 2.6. RT-qPCR Analysis

**Figure 11:**
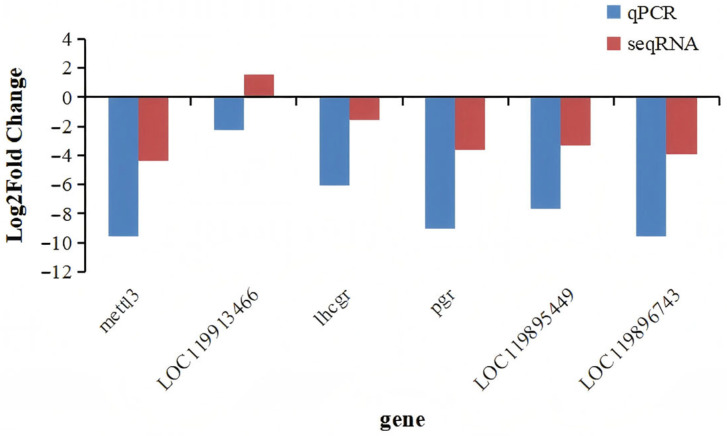
RT-qPCR verification results.

To verify the reliability of the sequencing results, six randomly selected genes were subjected to fluorescence quantitative PCR (RT-qPCR). The results indicated that the relative quantification trends were generally consistent with those obtained from RNA-seq, thereby confirming the reliability of the sequencing outcomes (Figure 11).

## 3. Discussion

### 3.1. Water Hyacinth and Water Qualities

Compared to traditional monoculture, the experimental model integrating water hyacinth into largemouth bass ponds triggered a series of beneficial changes within the ecosystem. The general finding is consistent with the established literature and underscores the efficacy of the plant in sustaining and enhancing the aquaculture ecological environment that is more conducive to fish growth. At the commencement of this experiment, the FM and M ponds exhibited similar water nutrient levels. However, the concentrations of total nitrogen (TN), total phosphorus (TP), and ammonia nitrogen (NH_3_-N) in the two groups diverged significantly (*p* < 0.05) over time, a trend that became particularly pronounced after the 60th day and persisted until the very end of this experiment (Table 1). By Day 90, the FM ponds exhibited a marked reduction in TN and TP, respectively, by 52% and 35% on average, relative to day 0, whereas the M ponds saw increases of 35% and 61% (Table 1), illustrating the cumulative and increasing efficacy of water hyacinth in nutrient removal over the growth period. The underlying mechanism involves the assimilation of nutrients, particularly nitrogen (N) and phosphorus (P), from the water column by water hyacinth through its stems and leaves, and the synthesis of its own organic components utilizing these nutrients [14,17].

Water transparency (TS) and DO also show significant differences between the two groups at both 60d and 90d (Table 1), with values of FM ponds substantially higher than those of M ponds. Although extensive studies have reported that high coverage of water hyacinth might decrease DO concentration [25,26], our study observed an increase in DO in ponds containing the plant, suggesting that a coverage rate of 20% may represent a safe and beneficial threshold for water hyacinth cultivation in fish farm environments. On the other hand, as was demonstrated by Schindler et al. [27], the reduction in both TN and TP under other controlled conditions could lead to dramatic drops of algal biomass and the proportion of cyanobacteria, resulting in prominent increases in transparency and DO. Meanwhile, with its extensive root systems and large leaves, water hyacinth can directly suppress the growth of other phytoplankton through nutrient competition [28] and reduce the concentration of suspended matter via interception and sedimentation functions [29], thereby decreasing turbidity and increasing transparency. Similarly, in our experiment, it is well-grounded to attribute the marked improvements in water transparency and DO in the FM ponds to the introduction of water hyacinth. Overall, the water quality results from this experiment further illustrated the effectiveness of water hyacinth, at a scientifically determined level, in removing excess nutrients and improving water quality in largemouth bass farming systems.

Nevertheless, due to its rapid growth and reproductive rate, proactive measures must be implemented to prevent the uncontrolled spread of water hyacinth. Without effective management, excessive proliferation of this plant would significantly deplete dissolved oxygen levels, diminish underwater light penetration, restrict the habitat available for farmed fish, and ultimately impair their growth and survival [19,30].

To proactively manage this risk in our study, we implemented a controlled cultivation design. An area equivalent to 20% of the total pond surface was allocated for water hyacinth. This area was subdivided into two zones (each 10% of the total area), demarcated along the long sides of the rectangular pond and physically isolated from the open water by perimeter ropes. This confinement strategy effectively prevented uncontrolled expansion throughout this experiment, thereby allowing us to study its benefits while mitigating its ecological risks. However, in practical aquaculture operations, these measures may not be easily implemented, which highlights the need to develop measures to effectively address the spread and negative consequences of water hyacinth.

### 3.2. Effects on Largemouth Bass Growth and Ovary Development

The above analyzed water quality changes directly or indirectly led to the observed differences in largemouth bass growth, with the FM group demonstrating a statistically significant 17% increase over the M group (Table 2). As the most notable differentiating factor throughout the latter half of this experiment between the FM and M groups, water transparency likely served as the primary driver behind these disparities. Miner & Stein [31] observed that under conditions of high water transparency and low turbidity, the predation success rate of largemouth bass on small bluegills could increase by 25–35%. Huenemann et al. [32] reported that increased turbidity levels impair the ability of largemouth bass to capture prey and prolong the time required for both locating and capturing prey. These collectively suggest that within the experimental conditions, the clearer water conditions in the FM group enhanced largemouth bass foraging efficiency, leading to better growth performance. Increases in growth associated with higher light intensity have also been reported in other photophilic fish species, including the southern flounder (*Paralichthys lethostigma*) and leopard coral grouper (*Plectropomus leopardus*) [33,34]. In addition, the significantly higher DO levels in the FM group, along with lower N and P concentrations, may also have contributed to the observed disparities in growth. As is well established, within a certain threshold, increasing DO often results in enhanced growth of fish, while hypoxia may limit their growth and physiological status [35,36,37,38,39]. For largemouth bass, it is recommended to keep the water DO at a high level as much as possible under achievable conditions to optimize metabolic efficiency [40].

Significant differences in GSI, a key indicator in evaluating fish gonad health and development, were also observed between the two groups in the current study, with the FM group exhibiting 17% elevation compared to the M group. Consistent with this observation, macroscopic morphology and histological analysis confirmed an enhanced level of ovarian development in largemouth bass fed the FM group in the present study. The ovarian histology of the FM group demonstrated typical morphological features of the secondary vitellogenesis (SVG) stage, which is a critical phase for substantial yolk accumulation. In contrast, the M group remained at the perinucleolar oocytes (PN) stage, suggesting that yolk accumulation had not yet initiated [41]. The better performance of FM group females in GSI and ovary development likely indicates superior nutritional intake as the major reason, and vice versa, while the M group may be associated with nutritional deficiencies [42,43,44], while indirectly, the differences in water environment (e.g., light condition and DO) may act as important contributing factors. Boeuf et al. [45] demonstrated that light acts as a key environmental factor regulating metabolic and endocrine processes in cultured fish, thereby affecting fish reproduction. Consistently, recent studies further indicate that the gonadal development and spawning in fish are influenced by photoperiod extension and light condition variations [46,47,48]. Research on largemouth bass has similarly found that prolonged light exposure enhances sex gland development [49]. However, its gonadal initiation also requires short-term light stimulation [41]. In the FM pond, water hyacinth profoundly increased the water transparency while also providing shade and facilitating short-term light stimulation, which may partially explain the enhanced ovary development observed in the FM group in the current study. Moreover, desirable DO levels, as in the FM group, promote gonad development by reducing metabolic maintenance cost and improving feed efficiency. In contrast, hypoxia adversely affects reproduction, causing disorders like follicle atresia and delayed oocyte growth [44,50]. In the present study, such developmental retardation was evident in the M group.

### 3.3. Metabolomics and Transcriptomic Analysis

Through metabolomic and transcriptomic profiling, we sought to explore at the molecular level the potential mechanisms underlying the observed differences in growth and ovarian development between the two groups.

Metabolomic profiling indicated a significant enrichment of the glycerophospholipid metabolism pathway in the FM group. As the predominant phospholipid class in cellular membranes, glycerophospholipids—particularly phosphatidylcholine (PC) and phosphatidylethanolamine (PE)—serve as essential structural components for membrane biogenesis. Elevated levels of these phospholipids are thus associated with enhanced cellular proliferation and tissue growth, which aligns with the improved physiological performance observed in the FM group in this study. In addition, PC contents in juvenile fish have previously been reported to correlate positively with weight gain rate, a finding consistent with the present study [51]. Moreover, the products of glycerophospholipid pathways, such as phosphatidic acid, can maintain the stability of cell membranes [52]. Glycerophospholipid metabolism also has the function of maintaining cell membrane fluidity, as well as participating in the energy metabolism of the organism [53]. The significant enrichment of the glycerophospholipid metabolism pathway in the FM group may elucidate, at a molecular level, mechanisms underlying the superior growth performance of largemouth bass fed the FM diet compared with the control. On the other hand, glycerophospholipids play a direct and crucial role in gonadal development and oocyte maturation of ovoviviparity teleosts [54]. This vital role of glycerophospholipids in reproduction is conserved across aquatic species. For instance, in Pacific white shrimp (*Litopenaeus vannamei*), enhanced nutrient accumulation (e.g., triglycerides) promoted ovarian development by modulating glycerophospholipid and key fatty acid metabolism [55]. It has also been reported that the metabolism of glycerophospholipids and sphingolipids has a significant impact on the sex differentiation and development of the Japanese marsh shrimp (*Penaeus japonicus*) [56]. Collectively, these findings suggest that there is a conserved and functionally significant link between glycerophospholipid metabolism and ovarian development across aquatic species. Our results in largemouth bass not only align with this established association but also provide further physiological validation of this mechanism.

The transcriptome analysis results further revealed the methyltransferase-like 3 (*Mettl3*) gene as the most markedly upregulated gene in the FM group, showing the highest fold-change in expression. As a methyltransferase responsible for RNA m_6_A modification, *Mettl3* is essential for early embryogenesis in zebrafish [57]. Studies have shown that the knockdown of *Mettl3* mirrored the effects of TCS exposure, adversely impacting the growth and development of zebrafish, as well as the differentiation of innate immune cells, demonstrating the promoting effect of the *Mettl3* gene on zebrafish growth [58]. Similarly, the enhanced *Mettl3* mRNA expression in the FM group aligned with the relationship between the gene and growth performance of largemouth bass in the current study. In addition, *Mettl3* drives vitellogenesis of fish juveniles by maintaining the mRNA stability of key reproductive genes, such as the bitellinogen gene (*vtg*), estrogen receptor gene (*erα*), and oocyte maturation factor (*bmp15*), and without the *Mettl3* gene (knockdown), ovary development stopped, or oocyte maturation halted at the primary yolk stage [58,59]. In the current study, the *Mettl3* gene exhibited the most pronounced differential expression, and it is hereby hypothesized that this gene plays an important role in regulating ovarian development in largemouth bass. The elevated *Mettl3* gene expression observed in the FM samples provides a putative molecular mechanism underlying their higher gonadosomatic index (GSI) and advanced ovarian development.

Furthermore, melatonin treatment can increase the total m_6_A levels in cells greatly, accompanied by remarkable expression elevation of the m_6_A writer [60]. Studies have found that light could affect melatonin production in fish; for example, light increases the activity and abundance of the AANAT expressed in trout retina, thus leading to Pineal melatonin synthesis increases at night and inhibiting fish gonadal development [61]. Wang et al. [62] found that changes in light intensity may lead to a decrease in the respiratory frequency of female *P. clarkii*, thereby diverting the energy allocated to respiration to growth and ovarian development. Therefore, in the present study, the gonadal differences between the FM and M groups may largely be attributed to the light differences caused by the tremendous transparency variations since the transparency of natural waters may be far more important in determining the quantity of light at a given depth than seasonal changes in the illumination reaching the surface of the water. According to Chandler [63], in certain waters the seasonal variation in illumination at the surface resulted in a 2-fold difference in the intensity of submarine light at 30 m., while a seasonal change in transparency caused a 1000-fold change at the same depth. The introduction of water hyacinth into the FM pond brought about notable alternation in water transparency and enhanced the light conditions, which then may have triggered endocrine changes that could be related to gonad development, resulting in the observed differences between the two groups.

Overall, co-culturing water hyacinth in a largemouth bass pond altered the ecological conditions of the living environment for the fish, resulting in a series of differences compared to the pond without water hyacinth. The differences were first exhibited in water quality parameter values, including TN, TP, DO, and particularly the transparency, followed by variations in growth performance (BW in particular) and female gonadal development, illustrating that the 20% ratio of water hyacinth planting could effectively improve pond water quality, benefit largemouth healthier growth, and promote ovary development. The metabolomics and transcriptomic analysis findings aligned with the apparent differences in that both the identified most enriched metabolic pathway (glycerophospholipids) and most notably enhanced gene expression (*Mettl3*) are closely related to fish growth and gonad development, offering a perspective for future studies to explore deeper how the external differences caused by planting water hyacinth resulted in the physiological differences among the fish individuals.

## 4. Materials and Methods

### 4.1. Experimental Design and Management

This experiment was conducted at the Yang Zhong experimental base of the Freshwater Fisheries Research Center, Chinese Academy of Fishery Sciences (N 32°31′, E 119°80′). Six ponds, each 0.17 ha earthen ponds, were used in a two-treatment design with three replicates each. Three ponds served as the control group (M group) without water hyacinth, while the other three constituted the water hyacinth integrated group (FM). In the FM group, water hyacinth was planted in clusters (1.5 m spacing) along both ends of the longer pond axis, covering 10% of the area at each end and 20% of the total pond surface (Figure 1). LBM fingerlings were sourced from Zhanglin Fishery Co., Ltd. (Tongling, China) and acclimatized for two weeks at the base. During acclimation, all fish were fed with the same commercial formulated diet (≥47% crude protein, ≥5% crude lipid; Xinxin Tianen Aquafeed, Jiaxing, China). After acclimation, fingerlings with an initial body weight of 14.50 ± 0.23 g were randomly selected and evenly stocked into the six ponds at a density of 43.48 g·m^−3^. The 90-day experiment lasted from 25 June 2022 to 25 September 2022, during which the fish were fed twice daily (08:00 and 18:00) to apparent satiation.

### 4.2. Water Quality and Growth Indicators

Water samples were collected from five fixed points in each pond on days 30, 60, and 90 of the trial. Dissolved oxygen (DO) was measured in situ using an HQ30D portable multimeter (HACH, Loveland, CO, USA). Total nitrogen (TN) and total phosphorus (TP) concentrations were determined using online water quality analyzers (LB-1000TN, LB-1000TP, respectively; Nanjing, China). Other physicochemical parameters were analyzed following international standard methods: Ammonia nitrogen (NH_3_-N) was measured using Nash’s reagent spectrophotometry, nitrite nitrogen (NO_2_^−^-N) by spectrophotometry [64], and nitrate nitrogen (NO_3_^−^-N) via the zinc–chromium reduction method. Water transparency was recorded as Secchi disk depth (SDD).

On the first day of this experiment, after a period of food deprivation, the initial body weight (BW_0_) and body length (BL_0_) of 18 randomly selected fish were recorded. On day 90 (25 September), 18 females were sampled from each group, and their body length (BL_t_) and body weight (BW_t_) were measured individually. Fish were then euthanized with MS-222 (50 mg/L) (Argent Chemical Laboratories, Redmond, WA, USA) and dissected to obtain the visceral fat and gonads for calculation of their weights and other body indices. Gonads were excised, weighed, and sectioned, and six pieces were immediately fixed in Bouin’s solution for histological analysis, while the remaining tissue was stored at −80 °C for metabolomics and transcriptome assays.

The gonadosomatic index (GSI), visceral somatic index (VSI), condition factor (CF), and specific growth rate (SGR) were calculated according to the following formulae:

GSI (%) = (gonad weight/W_t_) × 100

VSI (%) = (visceral weight/W_t_) × 100

CF (g/cm^3^) = W_t_/L_t_^3^

SGR (%/d) = (InW_t_ − InW_0_)/t × 100%

### 4.3. Gonad Histological Analysis

Based on the GSI results (Figure 2), the GSI values of the M group fell entirely within the range of 0.01–0.02, whereas those of the FM group ranged from 0.01 to 0.06, with 21.4% of individuals in the 0.01–0.02 interval, 48.1% in 0.02–0.03, and 30.5% in 0.03–0.06. For histological analysis, three ovarian samples were collected from the M group, all representing the 0.01–0.02 GSI range (labeled M1). From the FM group, three samples were selected from each of the three GSI intervals and designated as FM1 (0.01–0.02), FM2 (0.02–0.03), and FM3 (0.03–0.06). After fixation (>2 days), the selected gonad samples were block-trimmed, dehydrated through a graded alcohol series, cleared with xylene, embedded in paraffin, and sectioned serially at 7 μm. Sections were stained with hematoxylin and eosin (H&E), mounted with neutral balsam, and observed under a light microscope for imaging.

### 4.4. Gonad Metabolomics Analysis

For metabolomic and transcriptomic profiling, representative ovarian samples from groups M1, FM2, and FM3 were selected. Gonad tissues were ground in liquid nitrogen, and 100 mg aliquots were weighed for metabolite extraction. Each aliquot was homogenized with 200 μL of ice-cold water and 800 μL of pre-chilled methanol/acetonitrile (1:1, *v*:*v*). After thorough vortexing, samples were sonicated in an ice bath for 60 min and incubated at −20 °C for 1 h to precipitate proteins. Following centrifugation at 16,000 r/min for 20 min at 4 °C, the supernatant was collected for analysis. Chromatographic separation was performed on ultra-high-performance liquid chromatography (UPLC) with a HILIC column maintained at 25 °C. The injection volume was 5 μL, and the flow rate was 0.3 mL/min. The gradient elution program was as follows: 95% acetonitrile from 0 to 0.5 min, 95% to 65% acetonitrile (linear) from 0.5 to 7 min, 65% to 40% from 7 to 9 min, held at 40% from 9 to 10 min, 40% to 95% from 10 to 11.1 min, and a re-equilibration at 95% from 11.1 to 16 min. Throughout the analysis, samples were kept at 4 °C in the autosampler. Metabolites were detected using electrospray ionization (ESI) in both positive and negative ion modes coupled to a mass spectrometer.

### 4.5. Gonad Transcriptome Analysis

Total RNA was extracted from the tissue using TRIzol^®^ Reagent following the manufacturer’s protocol. RNA integrity and concentration were assessed with a 5300 Bioanalyser (Agilent Technologies, Beijing, China) and an ND-2000 spectrophotometer (NanoDrop Technologies, Wilmington, DE, USA), respectively. Only high-quality RNA samples (OD260/280 = 1.8~2.2, OD260/230 ≥ 2.0, RQN ≥ 6.5, 28S:18S ≥ 1.0, >1 μg) were used for subsequent library preparation.

RNA purification, reverse transcription, library construction, and sequencing were performed at Shanghai Majorbio Bio-pharm Biotechnology Co., Ltd. (Shanghai, China). Stranded mRNA-seq libraries were prepared using the Illumina^®^ Stranded mRNA Prep, Ligation kit (Illumina, San Diego, CA, USA) starting with 1 μg of total RNA per sample. Briefly, polyadenylated mRNA was enriched with oligo (dT) beads and fragmented. First-stranded cDNA was synthesized using the SuperScript double-stranded cDNA synthesis kit (Invitrogen, Carlsbad, CA, USA). The resulting cDNA underwent end-repair, phosphorylation, and adenylation according to the Illumina protocol. Libraries were size-selected f (~300 bp) on a 2% Low Range Ultra Agarose gel, amplified with Phusion DNA polymerase (NEB) for 15 PCR cycles, and quantified using a Qubit 4.0 fluorometer. Finally, paired-end sequencing (2 × 150 bp) was performed with on a NovaSeq X Plus sequencer (Illumina, San Diego, CA, USA).

The raw paired-end reads were trimmed and quality-filtered using fastp with default parameters. The resulting clean reads were then separately aligned to the reference genome in orientation-aware mode using HISAT2. Finally, the mapped reads from each sample were assembled with StringTie using a reference-guided approach.

Differential expression analysis was performed to identify differentially expressed genes (DEGs) between two sample groups. Transcript abundance was estimated using the transcripts per million reads (TPM) method and quantified with RSEM. Differential expression was assessed with DESeq2 or DEGseq, applying significance thresholds of |log2FC| ≥ 1 and FDR < 0.05 for DESeq2, or FDR < 0.001 for DEGseq. In addition, functional-enrichment analyses including Gene Ontology (GO) and Kyoto Encyclopedia of Genes and Genomes (KEGG) were performed to identify terms and pathways significantly overrepresented among DEGs compared to the whole-transcriptome background, using a Bonferroni-corrected *p*-value < 0.05 as the significance cutoff. GO functional enrichment was performed with Goatools, and KEGG pathway analysis was implemented using the SciPy library in Python (version Python 3.12).

All alternative splicing events in our samples were identified using latest release of rMATS. Only isoforms aligned to the reference or containing novel splice junctions were retained for analysis. Splicing differences were classified as exon inclusion, exclusion, alternative 5′ splice sites, alternative 3′ splice sites, and intron retention events.

### 4.6. Statistical Analysis

Data were organized using Microsoft Excel and analyzed with IBM SPSS Statistics v20. All results are presented as mean ± SD. A *t*-test was employed to assess the differences in various physical and chemical indices, with *p* < 0.05 considered statistically significant. Significant differences between groups are denoted by asterisks (* *p* < 0.05, ** *p* < 0.01, and *** *p* < 0.001).

## 5. Conclusions

In this experiment, the introduction of water hyacinth in the treatment group, as compared to the control group, triggered a cascade of ecological and physiological responses, which were manifested in measurable differences in water quality parameters (TN, TP, NH3-N, DO, and transparency), fish growth performance, gonadal development indices, and underlying metabolic/molecular pathways, including the gonad-related gene expression. Among the water quality indices, water transparency emerged as the most significantly altered parameter, a change driven by the plant’s combined effects on nutrient assimilation, surface shading, and oxygen dynamics. The underlying molecular mechanisms were closely associated with a pronounced enrichment of the glycerophospholipid metabolic pathway and a significant upregulation of the m^6^A methyltransferase core gene, *Mettl3*. The enriched glycerophospholipid metabolism supports enhanced cellular membrane biosynthesis and energy provision, aligning with observed improvements in somatic growth and oocyte development. Concurrently, the elevated *Mettl3* expression is consistent with a potential enhancement of m^6^A RNA methylation activity in the FM group. This aligns with the established role of *Mettl3* in post-transcriptional regulation, suggesting a plausible mechanism through which key growth and reproduction-related gene transcripts might be stabilized. Taken together, the altered light regime induced by water hyacinth likely acted as a key environmental signal driving the upregulation of growth- and reproduction-related genes in LMB. The 20% surface coverage of water hyacinth created significantly improved pond conditions, enhancing both growth and reproductive success in cultivated species, and offering a replicable framework for future large-mouth bass ecological farming operations.

Although the current study identified *Mettl3* as the most upregulated gene and discussed its established role in gonadal development, we did not experimentally validate the specific molecular pathway linking light-driven changes alter *Mettl3* expression and subsequent gonadal effects in largemouth bass. Thus, the precise mechanism by which light modulates gonadal development at the molecular level remains unclear and warrants further studies.

## Figures and Tables

**Table 1:** Physicochemical parameters of water in the control (M) and water hyacinth (FM) groups. Data are expressed as mean ± SD (*n* = 3). Significant differences between groups are indicated by asterisks (* *p* < 0.05, and *** *p* < 0.001).

Indicators	30 d		60 d		90 d	
	M	FM	M	FM	M	FM
TN (mg/L)	6.15 ± 1.23	6.59 ± 1.35	7.25 ± 1.28 *	2.57 ± 1.08	8.33 ± 1.02 *	3.16 ± 0.92
TP (mg/L)	0.38 ± 0.12	0.43 ± 0.18	0.987 ± 0.37 *	0.397 ± 0.16	0.61 ± 0.18 *	0.28 ± 0.10
SP (mg/L)	0.08 ± 0.00	0.10 ± 0.05	0.114 ± 0.05	0.095 ± 0.03	0.09 ± 0.02	0.05 ± 0.02
NH_3_-N (mg/L)	0.02 ± 0.01	0.03 ± 0.01	0.21 ± 0.07 *	0.08 ± 0.05	1.12 ± 0.78 *	0.21 ± 0.04
NO_2_^−^-N (mg/L)	0.01 ± 0.00	0.01 ± 0.00	0.02 ± 0.00	0.03 ± 0.01	0.04 ± 0.01	0.04 ± 0.02
NO_3_^−^-N (mg/L)	0.76 ± 0.27 *	0.35 ± 0.10	0.43 ± 0.10	0.51 ± 0.19	0.93 ± 0.12 *	0.51 ± 0.08
TS (cm)	40.12 ± 3.34	45.27 ± 6.51 *	35.69 ± 2.97	140.24 ± 11.26 ***	30.15 ± 8.99	165.24 ± 20.18 ***
DO (mg/L)	6.11 ± 0.26	7.22 ± 1.76	5.15 ± 0.97	9.85 ± 0.31 *	4.02 ± 1.08	9.22 ± 2.88 *

**Table 2:** Growth index of largemouth basses. Data are presented as mean ± SD (*n* = 3). Significant differences between groups are indicated by asterisks (* *p* < 0.05 and *** *p* < 0.001). BL: body length; BW: body weight.

Indicators	Time/d	M	FM
BL/mm	30	144.77 ± 8.19	152.15 ± 5.43
	60	181.60 ± 9.00	179.11 ± 11.04
	90	221.83 ± 11.87	219.30 ± 10.01
BW/g	30	67.72 ± 8.38	76.80 ± 9.24
	60	153.77 ± 16.72	143.66 ± 18.37
	90	305.31 ± 61.61	337.28 ± 46.96 *
SGR/%·d^−1^	90	1.67 ± 0.95	1.64 ± 0.89
CF/g/cm^3^	90	2.722 ± 0.281	3.184 ± 0.190 *
GSI/%	90	0.016 ± 0.003	0.039 ± 0.017 ***
VSI/%	90	0.107 ± 0.012	0.132 ± 0.019

## Data Availability

The raw sequence data are available at https://www.ncbi.nlm.nih.gov/sra/PRJNA1214894 (accessed on 28 January 2025).
